# Efficacy and safety of netupitant/palonosetron combination (NEPA) in preventing nausea and vomiting in non-Hodgkin’s lymphoma patients undergoing to chemomobilization before autologous stem cell transplantation

**DOI:** 10.1007/s00520-021-06495-0

**Published:** 2021-09-17

**Authors:** Nicola Di Renzo, Maurizio Musso, Rosanna Scimè, Alessandra Cupri, Tommasina Perrone, Clara De Risi, Domenico Pastore, Attilio Guarini, Andrea Mengarelli, Fabio Benedetti, Patrizio Mazza, Saveria Capria, Patrizia Chiusolo, Luca Cupelli, Vincenzo Federico, Valentina Bozzoli, Anna Rita Messa, Rosella Matera, Davide Seripa, Paolo Codega, Erminio Bonizzoni, Giorgina Specchia

**Affiliations:** 1 Hematology and Stem Cell Transplant Unit, “Vito Fazzi” Hospital, Lecce, Italy; 2 Hematology and Transplant Unit, Oncology Department La Maddalena, Palermo, Italy; 3 UTMO Vito Cervello Hospital, Palermo, Italy; 4 Ferrarotto Hospital, Catania, Italy; 5 Hematology Unit, Department of Emergency and Organ Transplantation, University of Bari, Bari, Italy; 6 Hematology and Transplant Unit, Cardinal Panico Hospital, Tricase, Italy; 7 Hematology Unit, A. Perrino Hospital, Brindisi, Italy; 8 Hematology Unit, Giovanni Paolo II IRCCS Cancer Institute Oncology Hospital, Bari, Italy; 9 USOD Hematology and Transplant Unit, Regina Elena IFO National Cancer Institute, Roma, Italy; 10 Department of Medicine, Section of Hematology and Bone Marrow Transplant Unit, University of Verona, Verona, Italy; 11 Department of Hematology-Oncology, Moscati Hospital, Taranto, Italy; 12 Department of Translational and Precision Medicine, Hematology Unit, Sapienza University, Policlinico Umberto I, Roma, Italy; 13 Hematology Department, Fondazione Policlinico Universitario Agostino Gemelli, Roma, Italy; 14 Hematology Unit, Sant’ Eugenio Hospital, Roma, Italy; 15 Medical Affairs Department, Italfarmaco SpA, Milan, Italy; 16 Section of Medical Statistics and Biometry GA Maccacaro, Department of Clinical Science and Community, University of Milan, Milan, Italy

**Keywords:** CINV, Netupitant, Palonosetron, NEPA, ASCT, Multiday chemotherapy

## Abstract

**Purpose:**

Prevention of chemotherapy-induced nausea and vomiting (CINV) is particularly challenging for patients receiving highly emetogenic preparative regimens before autologous stem cell transplantation (ASCT) due to the daily and continuous emetogenic stimulus of the multiple day chemotherapy. While studies have shown effective prevention of CINV during the conditioning phase with NK_1_ receptor antagonist (NK_1_RA)-containing regimens, there have been no studies evaluating antiemetic use during chemomobilization prior to ASCT.

**Methods:**

This multicenter, open-label, phase IIa study evaluated the efficacy of every-other-day dosing of NEPA administered during chemomobilization in patients with relapsed-refractory aggressive non-Hodgkin’s lymphoma. Eighty-one patients participated.

**Results:**

Response rates were 77.8% for complete response (no emesis and no rescue use), 72.8% for complete control (complete response and no more than mild nausea), 86.4% for no emesis, and 82.7% for no rescue use during the overall phase (duration of chemomobilization through 48 h after). NEPA was well tolerated with no treatment-related adverse events reported.

**Conclusion:**

NEPA, administered with a simplified every-other-day schedule, show to be very effective in preventing CINV in patients at high risk of CINV undergoing to chemomobilization of hematopoietic stem cells prior to ASCT.

## Introduction

Autologous stem cell transplantation (ASCT) after chemomobilization and stem-cell harvest is widely used as a potential curative strategy to treat non-Hodgkin’s lymphoma (NHL). The combination of high-dose chemotherapy with granulocyte colony-stimulating factor (G-CSF) support is a commonly used chemomobilization strategy prior to ASCT [1].

Prevention of chemotherapy-induced nausea and vomiting (CINV) is particularly challenging for patients receiving highly emetogenic preparative regimens before ASCT due to the daily and continuous emetogenic stimulus of the multiple day chemotherapy [2, 3]. Evidence-based antiemetic guidelines now recommend co-administration of a combination regimen consisting of a neurokinin-1 (NK_1_) receptor antagonist (RA), 5-hydroxytryptamine-3 (5-HT_3_) RA, and dexamethasone (DEX) to prevent CINV in patients who are undergoing conditioning regimens for SCT [4, 5]. However, the guidelines are remiss in providing specific recommendations on the schedule of these agents in this setting beyond citing aprepitant studies where aprepitant was administered daily during and after the preparatory regimen [6–8]. In addition, the guidelines are focused on the conditioning phase prior to ASCT and do not provide any guidance for antiemetic regimens to be used during chemotherapy for mobilization of hematopoietic stem cells.

NEPA is a fixed combination antiemetic uniquely comprised of netupitant, a highly selective NK_1_ RA [9] and palonosetron, a second-generation 5HT_3_ RA, with a higher receptor affinity compared with first generation 5HT_3_ RAs [10]. Both molecules have an extended half-life (palonosetron 40 h and netupitant 90 h) relative to other agents in their classes. By simultaneously targeting two critical antiemetic pathways, NEPA offers a simpler more convenient antiemetic with the potential for long-lasting protection from CINV [11]. Oral NEPA plus DEX has shown superiority over oral palonosetron plus DEX for all key efficacy endpoints during the overall (0–120 h; 5 days) phase following single-day cisplatin- or anthracycline/cyclophosphamide (AC)-based highly emetogenic chemotherapy (HEC) [12, 13]. However, until recently, trials investigating the efficacy and the safety of NEPA in chemomobilization settings were not available.

The rationale of the current study was to explore the efficacy and the safety of NEPA in preventing CINV in patients with non-Hodgkin’s lymphoma (NHL), eligible for ASCT and treated with chemomobilization followed by preparative regimens for ASCT. Results of the conditioning phase of the current study were previously published [14]. The administration of every-other-day NEPA without the addition of DEX was found to be well-tolerated and very effective in controlling both emesis and nausea in patients at high risk of CINV undergoing FEAM/BEAM-based conditioning regimen.

This study differentiates itself from prior trials of aprepitant in this setting. Given the long half-lives of both netupitant and palonosetron, NEPA was administered in the study as an every-other-day regimen, as opposed to daily as in the aprepitant trials. Notably, prior aprepitant studies were performed in combination with DEX as part of the antiemetic regimen [6–8]. However, as DEX exhibits immunosuppressive activity and can result in adverse events, including serious infections in patients undergoing hematopoietic stem cell transplantation, NEPA was administered without DEX in this study, as in previous palonosetron studies in similar settings [15, 16].

To our knowledge, this is the first study designed to explore the antiemetic efficacy of an NK_1_ RA regimen during the mobilization phase as well as the conditioning phase during which multiple day chemotherapy is administered for mobilizing stem cells prior to ASCT. Prior studies have only evaluated aprepitant/fosaprepitant during the conditioning phase [17, 18]. The intent of this paper is to present the efficacy and safety of NEPA in preventing CINV associated with the emetogenic multiple drugs administered during the mobilization phase of this study.

## Methods

### Study design

This was a Phase IIa, open-label, non-comparative study with a single-stage Fleming design conducted between January 2016 and February 2018 in 28 Italian centers. Each study center obtained approval from the local institutional review board/ethics committee and all patients provided written informed consent prior to study enrollment.

### Patients

Eligible patients were ≥ 18 years old with a diagnosis of relapsed/refractory aggressive NHL and eligible for ASCT. Patients were to be appropriate candidates to receive multiple day salvage/mobilization chemotherapy followed by a preparative regimen of BEAM or FEAM prior to ASCT. After enrollment, the patients underwent the mobilization phase. Specific chemomobilization protocols were at the discretion of individual sites and physicians and therefore, consisted of a variety of regimens from 2–5 days duration. All regimens included chemotherapy agents that are classified as moderately to highly emetogenic according to antiemetic guideline classifications [4, 5]. Granulocyte-colony stimulating factor (G-CSF) support was utilized with or without plerixafor and peripheral collection of autologous CD34 + circulating stem cells by leukapheresis. After hematopoietic stem cell collection and eligibility for ASCT was confirmed, the patients entered the conditioning phase of the study.

### Treatment

During the mobilization phase NEPA was administered every other day of chemotherapy regimen, starting from the first day of chemotherapy administration, with a maximum of 3 total doses given in the case of a 5-day regimen. Use of dexamethasone for antiemetic prophylaxis was not allowed also in patients who did not receive it as a part of chemotherapy regimen, in order to decrease the risk of serious infections in these patients who were already heavily immunosuppressed. Oral metoclopramide (at a maximum dose of 30 mg/day) was used as a rescue antiemetic, if needed.

### Assessments

Nausea and vomiting were self-recorded by patients using a study diary, in which each episode of emesis, any use of rescue medication, the maximum grade of nausea according to the Likert scale (none, mild, moderate, and severe), and occurrence of any adverse event were recorded daily from Day 1 of chemomobilization until 48 h after the last dose of chemotherapy. At the end of the observation period, the patients’ global satisfaction with the CINV prophylaxis was also collected by means of a visual analog scale (VAS) from 1 to 10. Treatment-emergent adverse events (TEAEs) were also monitored and recorded during the study according to the Common Terminology Criteria for Adverse Events version 4.3.

### Statistical analysis

The primary objective of the study was to evaluate the complete response (no emesis and no rescue medication) rate during the overall period of the mobilization phase, defined as from Day 1 of chemotherapy until 2 days after the last dose of chemotherapy. The pre-defined assessment of the efficacy of NEPA for prevention of CINV during the mobilization phase was a secondary objective. Efficacy endpoints for this analysis included complete response (no emesis, no rescue medication), complete control (complete response with a maximum grade of mild nausea), emesis-free (no emesis), rescue-free (no rescue medication), nausea severity, and patient global satisfaction.

Efficacy endpoints were evaluated from Day 1 until 48 h after the last dose of chemotherapy (i.e., the overall phase) for each patient, as well as, during the acute phase (from Day 1 until last dose of chemotherapy) and delayed phase (from last dose until 48 h after the last dose). Response was also assessed on each individual day during the overall phase and summarized for the study population. For each endpoint, results were summarized with cumulative incidences with associated two-tailed 95% exact binomial confidence intervals.

## Results

### Patient population

A total of 82 patients were screened and included in the enrolled population; 81 of these were included in the mobilization analysis (one patient was excluded for undergoing an allogeneic transplantation). Qualitative and quantitative demographic characteristics are summarized in Table 1 and are consistent with the target population of the study. Mobilization included a variety of regimens with or without rituximab. Optimal mobilization was achieved in 97.5% of patients, with the majority of patients (90.1%) undergoing only one apheresis procedure.

**Table 1:** Summary of patients’ characteristics (enrolled population)

Age (years)	
Mean ± SD	54.04 ± 10.77
Median (25th–75th)	56.5 (48–62)
Min–Max	24–78
Gender	
Male	51 (62.2%)
Female	31 (37.8%)
ECOG	
0	48 (58.5%)
1	29 (35.4%)
2	5 (6.1%)
Histology	
DLBCL	77 (93.9%)
MCL	2 (2.4%)
PMBCL	1 (1.2%)
PTCL	2 (2.4%)
Status of disease at study entry	
Relapsed	80 (97.6%)
Primary refractory	2 (2.4%)
No. previous therapy	
1	82 (100%)
Duration of mobilization regimen	
2 days	58 (70.7%)
3 days	21 (25.6%)
4 days	1 (1.2%)
5 days	2 (2.4%)
Optimal mobilization	80 (97.5%)
No. of apheresis	
1	74 (90.2%)
2	8 (9.7%)
Conditioning regimen	
FEAM	46 (56.1%)
BEAM	23 (28.0%)
Melphalan/Mitoxantrone	1 (1.2%)
None	12 (14.6%)

Legend: *DLBCL*, Diffuse Large B-Cell Lymphoma; *MCL*, Mantle Cell Lymphoma; *PMBCL*, Primary Mediastinal B-Cell Lymphoma; *PTCL*, Peripheral T-Cell Lymphoma.

### Efficacy

**Fig. 1:**
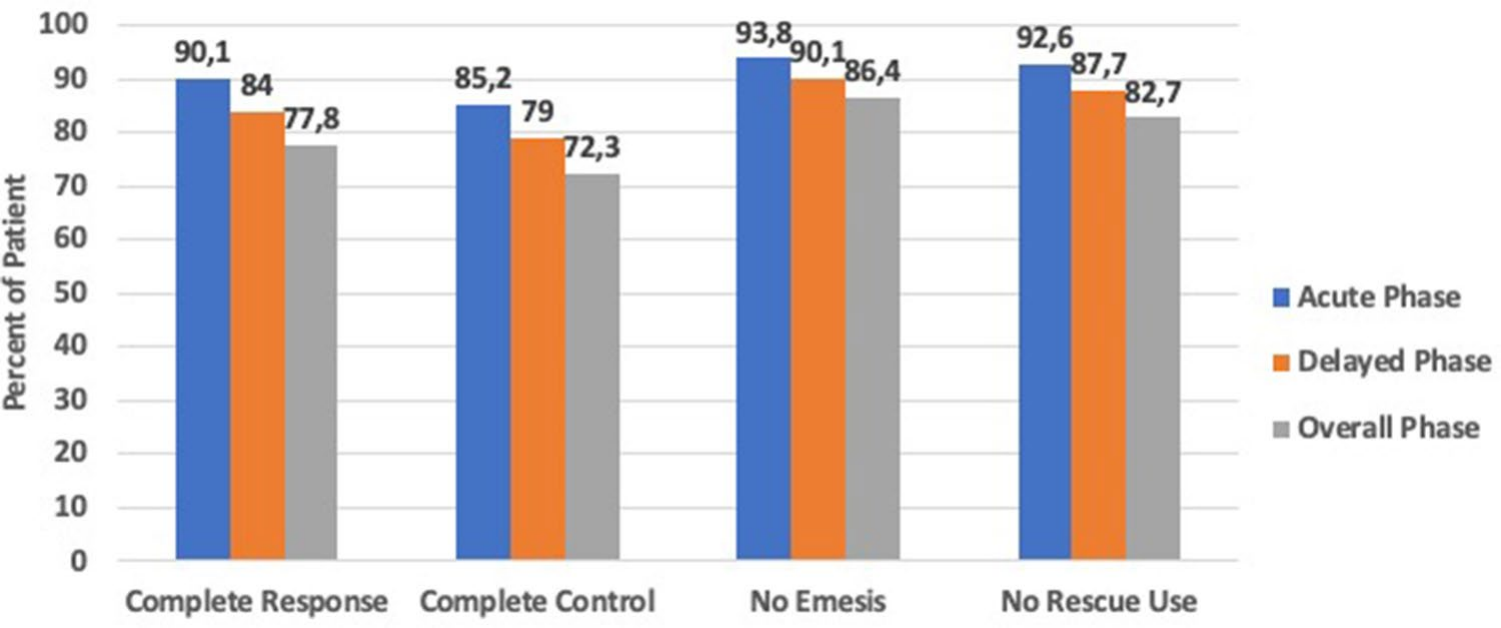
The histograms show the proportions of patients with complete response (no emesis and no rescue medication), complete control (complete response with no more than mild nausea), no emesis, and no rescue medication during the acute (from Day 1 until last day of chemotherapy), delayed (from last day of chemotherapy until 48 h after last dose of chemotherapy), and overall (from Day 1 until 48 h after last dose of chemotherapy) phases during mobilization

NEPA was shown to be highly effective in preventing CINV during the mobilization period, particularly for the endpoints of no emesis and no rescue use where overall phase rates were 86.4% and 82.7%, respectively. The proportions of patients with complete response and complete control during the overall phase were 77.8% and 72.8%, respectively (Fig. 1). Daily rates for each of these endpoints were consistently above 80% on each of the individual days during chemomobilization and during the 48 h after the final dose of chemotherapy.

**Fig. 2:**
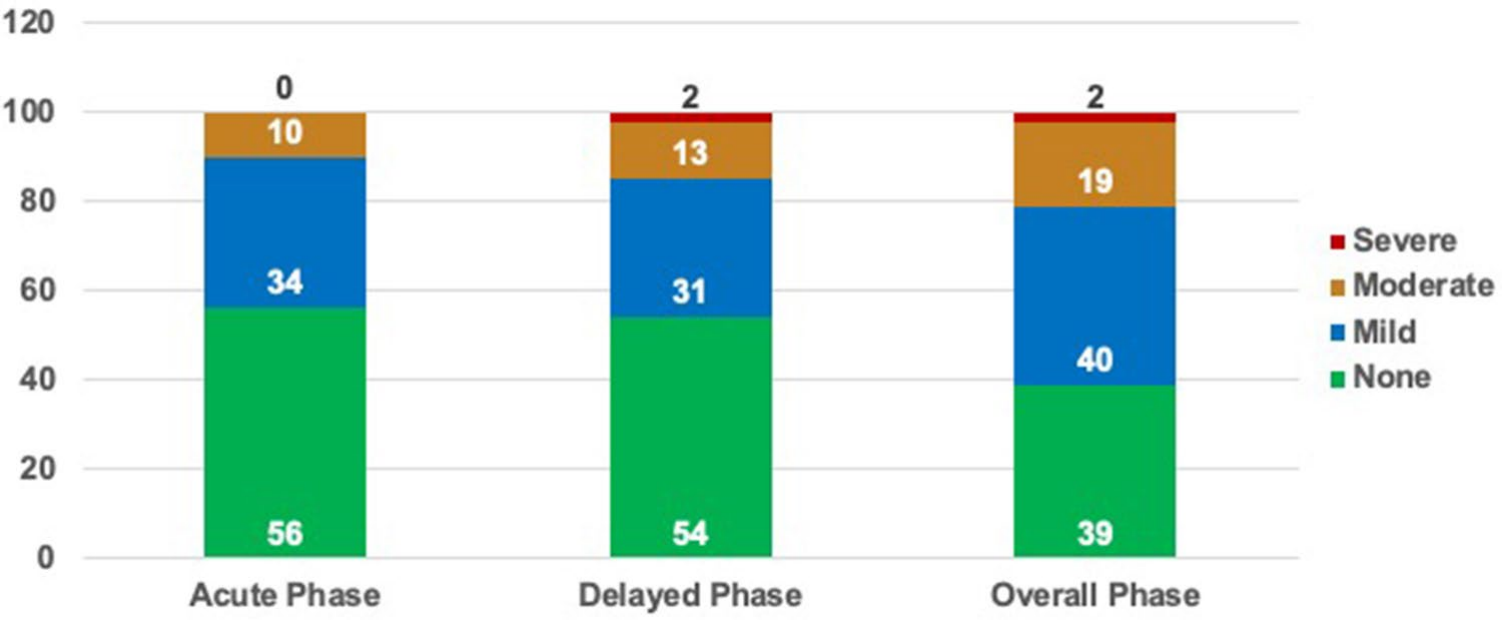
The histograms show the proportions of patients with no, mild, moderate or severe nausea during the acute (from Day 1 until last day of chemotherapy), delayed (from last day of chemotherapy until 48 h after last dose of chemotherapy), and overall (from Day 1 until 48 h after last dose of chemotherapy) phases of the mobilization period

Nausea severity graded on a 4-point Likert scale was also documented by patients. The majority of patients reported no nausea or only mild nausea during the acute, delayed and overall phases (Fig. 2). Severe nausea was only reported by 1 patient (1.2%) during the delayed and overall phases. The mean patient global satisfaction with the antiemetic efficacy of NEPA during the mobilization phase was 8.90 ± 1.56 out of 10.

### Safety

NEPA was very well tolerated, with only 10 TEAEs occurring in five (6.2%) patients during the mobilization phase of the study. None of these events was evaluated as possibly related to NEPA administration. Two TEAEs (both sepsis) deemed to be unrelated to NEPA were graded as severe, and three unrelated TEAEs (two sepsis and one fatigue) were classified as serious adverse events (SAE).

## Discussion

Despite significant advances in the management of CINV associated with single-day highly and moderately emetogenic chemotherapy, prevention of nausea and vomiting in patients with hematological malignancies has remained a challenge. In this setting, treatments are intense and elicit a persistent emetic stimulus, given that chemotherapy is generally administered repeatedly over multiple consecutive days [19, 20]. Even with a triple NK_1_RA/5-HT_3_RA/DEX regimen with components administered daily, response rates in the aprepitant/fosaprepitant studies are inconsistent [6–8, 17] and suggest a continued unmet need and opportunity for improved control of CINV, particularly nausea. The results seen in the conditioning phase of our study with NEPA more conveniently administered every-other-day were very encouraging, where a complete response (no emesis and no rescue use) rate of 87% was shown in the overall phase [14]. In addition, nausea was well controlled, with 93% of patients experiencing no or no more than mild nausea during the overall phase, well above the rates seen in prior aprepitant trials [6–8].

These findings are particularly impressive as this is the first study exploring the efficacy of NEPA in the setting of peripheral progenitor cells mobilization without use of DEX other than included in the chemotherapy regimen. While directly proportional to the total DEX dose and duration administered, the extent of corticosteroid-induced immunosuppression is impossible to predict. In the setting of ASCT, patients undergoing high-dose chemotherapy may develop deep, although transient, immunosuppression which can put the patient’s life at risk.

In this case and since some mobilization regimens contain DEX, any additional immunosuppression could lead to an increased risk of infection. This is the reason why the study does not include the administration of DEX for the control of nausea and vomiting induced by regimens that already contain it.

It is noteworthy that the study was conceived in 2015 when there were no guidelines for controlling CINV in this setting of patient. Only recently, NCCN updated guidelines (Version 1.2021) suggesting that DEX should not be added when the anticancer regimen already contains corticosteroids.

Although approximately 70% of patients in the study received a DEX-containing mobilization regimen, no statistically significant difference was observed in terms of complete responses, complete control, no nausea and rescue medication compared to those who did not receive DEX (Data not shown).

To our knowledge, this is also the first study to explore an antiemetic regimen in preventing CINV associated with multiple day chemotherapy being administered for mobilization of hematopoietic stem cells prior to ASCT. The most common mobilization regimen utilized in this study (DHAP/R-DHAP) is consistent with customary CT regimens administered and recommended by guidelines for this setting [1]. Consistent with the findings during the conditioning phase, response rates with every-other-day dosing of NEPA during mobilization were high, exceeding 85% for all efficacy endpoints during chemotherapy administration (acute phase) and ranging from 79 to 90% across endpoints during the 48 h following chemotherapy (delayed phase). Emesis control was excellent with 94% and 90% of patients experiencing no emesis during the acute and delayed phases, respectively. NEPA effectively controlled nausea as well, with the majority of patients (~ 85%) experiencing no or only mild nausea during the acute and delayed phases.

In conclusion, NEPA was found to be very effective in controlling both emesis and nausea in patients at high risk of CINV undergoing for mobilization of hematopoietic stem cells prior to ASCT. NEPA, as a long-acting highly effective combination antiemetic, simplifies dosing with an every-other-day schedule while also eliminating the use of corticosteroids in these heavily pretreated and immunocompromised patients.

## Data Availability

Yes.
